# Effects of insulin on placental, fetal and maternal outcomes in gestational diabetes mellitus

**DOI:** 10.12669/pjms.302.4396

**Published:** 2014

**Authors:** Rabia Arshad, Nasim Karim, Jahan Ara Hasan

**Affiliations:** 1 Dr. Rabia Arshad, MBBS, M.Phil (Pharmacology), Senior Lecturer, Pharmacology Department, Bahria University Medical and Dental College, Karachi, Pakistan.; 2 Dr. Nasim Karim, MBBS, M.Phil, Ph.D, Post Doc (Pharmacology), Head of Pharmacology Department, Bahria University Medical and Dental College, Sailors Street, Adjacent PNS Shifa, Defence Phase 2, Karachi, Pakistan.; 3 Dr. Jahan Ara Hasan, FCPS, MCPS, MBBS, Associate Professor, Obstetrics and Gynecology Department, Dow University of Health Sciences, Karachi, Pakistan.

**Keywords:** Gestational diabetes, Insulin, Placenta, Gross morphology, Fetal outcome, Maternal outcome

## Abstract

***Objective: ***To observe the effects of exogenous insulin on placental, fetal and maternal outcomes in Gestational Diabetes Mellitus (GDM).

***Methods: ***After screening and diagnoses(WHO criteria) 30 GDM patients(Group A) were kept on diet control and 39 GDM (Group B) who did not achieve glycemic targets were added subcutaneous insulin. Term placental weight, size, shape, consistency, fibrinoid necrosis, hemorrhages, cord color, length of the cord, completeness of membranes, weight and condition of baby and mode of delivery were assessed in 25 patients in each group.

***Result: ***Placental weight, cord width and baby weight were found to be more in Group B, than Group A and were statistically significant with p value 0.005, 0.02 and 0.003 respectively. Ten patients in group A and 17 patients in group B had cesarean deliveries.

***Conclusion: ***Exogenous insulin produces significant effects on the placental, fetal and maternal outcomes in patients with GDM

## INTRODUCTION

“Gestational Diabetes is defined as any degree of glucose intolerance with onset or first recognition during the pregnancy”. In pregnancy insulin sensitivity decreases, thereby pregnant females are at greater risk to have deranged blood glucose levels and subsequently some of them develop gestational diabetes mellitus (GDM). This occurs in 3-9% of pregnancies and is growing in prevalence^1^^,^^2^ WHO criteria is the most preferred criteria for GDM diagnosis, followed by Oral Glucose Tolerance Test for confirmation.^3^ If left untreated, GDM has effects on placenta, fetus and mother. According to modified Pedersen hypothesis, when hyperglcemic blood is carried to the fetus thru placenta, fetal pancreas releases large amounts of insulin leading to fetal hyper-insulinemia. This increased endogenous insulin acts as growth factors for fetus leading to storage of excessive amounts of glucose as glycogen and fat in the fetal body making these babies larger than the normal. Due to large sized fetus oxygen demand increases causing hypoxic condition in utero. This has direct and indirect effects on placenta leading to structural and functional alterations.^4^^,^^5^ Human placenta has a complex vascular system that allows exchange of different materials between fetal and maternal blood without actual mixing of the two. The successful development, growth and maturity of feto-placental vessels are important for normal fetal growth and survival.^6^^,^^7^ Complications of GDM encountered in fetus are increased birth weight, birth trauma, respiratory distress syndrome (RDS), hypoglycemia, hyperbilirubinemia, polycythemia, hypocalcemia, major congenital anomalies, intrauterine deaths at term and even still births where as in mother there are more chances of excessive weight gain, pre-eclampsia, cesarean sections and development of Type 2 diabetes in subsequent years.^8^

Blood glucose levels in the mother can be controlled by nutritional therapy (diet control) and exercise but in uncontrolled cases, where target glycemic levels could not be achieved medication are also required. Subcutaneous insulin is the traditional therapy and gold standard under such circumstances.^9^ Even with this pharmacotherapy, fetal and maternal morbidity and mortality are well documented in the literature^.^^10^ Morphological study of placenta which occupies central position between the mother and fetus might be helpful in elucidating these adverse fetal and maternal outcomes in gestational diabetes. With this background present study was designed to observe the effect of exogenous insulin on the gross morphology of placenta, fetal and maternal outcomes in gestational diabetics in our setting.

## METHODS

 This non-randomized clinical trial was conducted at Lyari General Hospital and Mamji Hospital Karachi in a period of 9 months. The study was approved by Ethical Review Board (ERB) and Institutional Review Board (IRB) of Dow University of Heath Sciences. Screening of high risk patients (High risks for GDM include patients with the history of GDM in previous pregnancies, females having babies large for gestational ages in previous pregnancies, history of still births, preterm delivery, recurrent abortions in previous pregnancies and females with strong family history of diabetes)^11^ was done in antenatal diabetic clinics and diagnosis was established according to WHO criteria. A written informed consent was taken from all the participants before enrollment in the trial. Initial screening was done with 50 gm oral glucose challenge test (OGCT, positive if ≥140mg/dl) in antenatal clinics, followed by 75 grams, 2 hours oral glucose tolerance test (OGTT) for confirmation. At least two abnormal readings, FBS > 95, 1 hr ≥ 180, 2 hr 155 mg/dl is diagnostic for GDM.^12^ Women between 18-45 years of age, diagnosed as GDM, having singleton pregnancy with no other co morbid were enrolled. Maternal demographic details (weight and age), FBS and RBS were noted in the start of the study on predesigned data form. These patients were kept on diet control for a week then their RBS was checked. Thirty patients with RBS between 126-129mg/dl were kept in Group A with the nutritional (diet control) and exercise therapy. They were advised and taught to take 2000-2500 kcal/day and special diet charts were provided. They were also advised to do 30 minutes of mild exercise (walk) thrice weekly. In Group B, thirty nine diagnosed GDMs with RBS more than 200 mg/dl were enrolled. They were given s/c insulin therapy (2/3 NPH + 1/3regular) according to the weight of the patient (0.8 units/kg/day in 2^nd^ trimester and 0.9units/kg/day in 3^rd^ trimester).^13^ The total dose of the drug was divided so as to take 2/3 of the drug in the morning and 1/3 drug in the evening. They were taught regular glucose monitoring and later the dose of the drug was adjusted accordingly with the targeted glucose level of FBS <100mg/dl and post prandial glucose ≤ 126mg/dl. The patients were followed in the antenatal clinics twice a month up till 36 weeks of pregnancy and then weekly till term. After childbirth the placentae were collected from twenty five patients in each group (A & B) who completed the study and delivered babies in the above mentioned hospitals. Within 30-40 minutes of delivery placentae were preserved in 10% formalin in labeled containers of adequate sizes, and then transferred to Dow Diagnostic Reference and Research Lab (DDRL). Gross morphological evaluation of placenta including placental weight, size, shape, consistency, membranes, any obvious deformity color and length, width and insertion of the umbilical cord was done. Feto-maternal outcomes including condition of the baby, weight of the baby and mode of delivery were noted down at the time of delivery. Data was analyzed by SPSS version 16. P value of 0.05 or less was considered statistically significant for the results. For the numerical variables mean with Independent t test and for categorical variables percentages with chi square tests were applied for evaluation. Fisher exact test was used where chi square test was not applicable due to decreased cell counts.

## RESULTS

Results are documented for 50 patients, 25 in each group who completed the study. Mean age of the patients was 30.08± 3.16 and 31.60 ± 4.27 years, mean weight was 78.54± 6.93 and 77.9±9.03kgs in GROUP A and Group B respectively, all being statistically non- significant. However FBS and RBS showed statistically significant results after one week of enrollment (Table-I).

Placental weight and cord width were more in Group B with significant p value of p=0.005 and p=0.02 respectively. Placental length, placental breadth, placental width, cord length, placental shapes, consistency, membranes completeness, cord insertion, cord color was statistically non-significant between the groups. More gross pathologies were found in Group B placentae (16/25) when compared with placentae in Group A (12/25). (Table IIa and IIb)

On comparison of fetal outcome, mean neonatal birth weight was more in Group B with p=0.003. Out of 25, 24 babies in group A and 23 babies in group B were healthy and born alive. There was one intrauterine death at term in Group A and 2 intrauterine deaths at term in group B.(Table-III)

In maternal outcomes, 15 (60%) mothers delivered normally, and 10 (40%) had cesarean sections in Group A. In Group B 8(32%) females delivered normally and remaining 17(68%) delivered by cesarean section due to big babies (Table-III).

## DISCUSSION

In our results statistically non-significant differences were present in between the two groups, in mean maternal age and mean maternal weight and same is documented by Dabella D in her study.^14^ FBS and RBS after 1 week indicate the WHO criteria for allocation and grouping of the patients. 

**Table-I:** Maternal characteristics. (Group A v/s Group B (n=50)).

*Variables*	*Group A (n=25)*	*Group B (n=25)*	*Significance*
	*mean± SD*	*mean± SD*	*p-value*
Patient Age (years)	30.08±3.16	31.60±4.27	0.159
Patient Weight (kg)	78.54±6.93	77.9±9.03	0.78
FBS	90.96±16.84	117±29.0	0.00*
RBS	123±38.9	239±69.7	0.00*

**Group A: **GDM on Diet and exercise control, **Group B: **GDM on Insulin treatment

FBS: Fasting blood sugar at OPD visit after 1 week of enrollment,

RBS: Random blood sugar after 1 week of enrollment, Student’s t test applied,

* statistically significant

**Table-IIa:** Gross examination of diabetic placentae. (Group A v/s Group B (n=50)).

*Numerical Variables*	*Group A (n=25)*	*Group B (n=25)*	*Significance*
	*mean± SD*	*mean± SD*	*P=value*
Placental weight(gm)	590 ± 147.9	698± 107.5	0.005*
Placental size length(cm)	15.06± 2.41	16.68 ± 3.43	0.06
Placental size breadth (cm)	12.88± 2.97	13.5± 2.45	0.41
Placental width(cm)	2.84± 0.62	2.48± 0.977	0.07
Cord length(cm)	42.96± 7.4	41.92± 7.0	0.61
Cord width(cm)	1.42± 0.55	1.84± 0.341	0.02*

**Group A: **GDM on Diet and exercise control, **Group B: **GDM on insulin treatment.

Student’s t test applied,

* statistically significant difference

**Table-IIb:** Gross examination of diabetic placentae. (Group A v/s Group B (n=50)).

*Categorical Variables*	*Group A (n=25)*	*Group B (n=25)*	*Significance*
Placental shape:			
Disc like Non-disc like	19 6	18 7	0.74
Placental consistency:			
Soft Hard	16 9	17 8	0.76
Cord insertion:			
Central peripheral	8 17	9 16	0.76
Cord Colour:			
Pale Blue	17 8	10 15	0.55
Membranes:			
Complete incomplete	22 3	19 6	0.43^
Obvious gross pathology in placental parenchyma:			
Hemorrhages Fibrinoid-necrosis Both necrosis and hemorrhages No gross lesion Other pathology	2(8%) 2(8%) 5(20%) 13(52%) 3(12%)	2(8%) 3(12%) 4(16%) 9(36%) 7(28%)	NA

**Group A: **GDM on Diet and exercise control. **Group B: ** GDM on insulin treatment.

Chi square test applied,

* Statistically significant,

^ Fisher exacts test applied as Chi square test not applicable due to less cell countsNA Chi square and fisher exact test not applicable

**Table-III:** Fetal and maternal outcomes. (Group A V/S Group B (n=50)).

*Variables*	*Group A (n=25)*	*Group B (n=25)*	*Significance*
Weight of the baby (kg)	3.09±0.3	3.44±0.46	0.003*
Condition of the baby			
Alive baby IUD Still births	24 (96%) 1(4%) 0	23(92%) 2(8%) 0	
Mode of delivery			
Normal vaginal Cesarean section	15(68%) 10(40%)	8(32%) 17(68%)	

Group A: GDM on Diet and exercise control. Group B: GDM on insulin treatment

Chi square test and students T test applied accordingly.

* Statistically significant.

Gestational diabetes produces changes in placenta secondary to change in the milieu of the mother and the fetus. To compensate the hyperglycemic blood from the mother, there is islets cell hypertrophy and beta cell hyperplasia of fetal pancreas with the release of excessive amounts of insulin in the fetal body. This results in a hyper-insulinemic state in the fetus with the up regulation of many genes expression, inflammatory mediators and leptin in placental tissues. This whole process probably produces excessive growth and increase in placental weight. Increased placental volume compensates the need of growing babies to an extent and after that hypoxic state generates leading to adverse fetal and maternal outcomes even unexplained termed intrauterine deaths. Placental tissues are liable to change with maternal metabolic issues. Our results have shown increase in placental weight in both the groups but a significant increase in placental weight is observed when glycemic control was done with diet control plus injectable insulin as compared to diabetics controlled on diet and exercise only. This indicates that exogenous insulin has exerted more effects on the placenta than the other group. According to Boyd, placental parenchymal tissues of insulin treated patients were much heavier in volume.^15^ Mayhew and Chowdhury worked on insulin treated diabetic pregnancies and found that GDM placental weights were significantly increased. The results of both these studies are in favor of our findings.^16^^,^^17^ Placental cord length, membrane completeness, placental shape, consistency, cord insertion, cord color and gross pathologies showed non-significant results between the two groups but with more propensities in the insulin treated group. Verma has discussed that on major gross examination of placentae, there were non-significant differences which is just similar to our results.^18^ It has been documented in literature that acute pulsatile rise and fall in the mother’s blood sugars as after food intake (hyperglycemia) and insulin treatment (hypoglycemia) might be unnoticed and account for increased fetal release of endogenous insulin resulting in big placenta and babies as compared to females on diet restricted therapy which is again in favor of our study.^19^

Our results have also shown that in insulin treated diabetic placentae, the cord width was significantly more but no literature support has been found in this regard. The relevance of these factors with the diabetic environment is yet to be evaluated but the probable reason of excessive growth of the cord tissue which makes it thicker is the effect of fetal insulin. Although non-significant statistically but more fibronoid necrosis was seen in insulin treated placentae. As per literature this type of necrosis is indication of placental compromise and therefore decreased supply of nutrients and oxygen to the growing fetus leading to hypoxia.^20^

When fetal outcomes were compared, it was seen that babies were much heavier in patients in insulin treated GDM group as compared to diet controlled group. Odar has stated that increased fetal weights leads to bad maternal and fetal outcomes, which is again similar to our study and the reason behind is the hyper-insulinemic state of the fetus affecting both the placental and fetal growth.^21^ Jansson described that probably excessive fetal growth is the result of increase in substrate availability which stimulates fetal insulin secretion and fetal growth. However, despite of strict glycemic control in modern clinical management of the pregnant woman with significant hyperglycemia, fetal overgrowth remains an important clinical problem. Recent studies have provided enough evidence for increased delivery of amino acids to the fetus in gestational diabetes (GDM), even when metabolic control is strict. So might be for this reason even when truly normal maternal substrate levels are achieved in diabetic pregnancies, the defect lies in altered placental nutrient transport and metabolism.^22^

Persson stated that babies of GDM mothers on insulin treatment and on diet control are similar in the weight.^23^ But a recent study by Wong has proven the fact that the babies of insulin treated GDM were heavier than diet control GDM mothers, so more GDM mothers on insulin treatment delivered through cesarean section due to fetal overgrowth and heavy term babies^24^ and is coinciding with our findings. We observed more intrauterine deaths in insulin treated group and the probable reason is the excessive growth of fetus which increases the oxygen demands. Placenta tries to compensate this to an extent but when the baby is grown enough and is near term, it cannot fulfill the requirements of fetus resulting in unexplained term intrauterine death in these patients.^25^ Our findings suggest that exogenous insulin probably improves the glycemic values but is unable to control the related problems completely as is evident from statistics. This point towards the presence of unknown areas in GDM pathology and need of alternative pharmacotherapy for GDM patients.

## CONCLUSION

Insulin has produced significant effects on the placental, fetal and maternal outcomes in patients having gestational diabetes mellitus in comparison to GDMs controlled on diet and exercise.

## Limitations of the study:

1. Following the patients throughout the pregnancy in the diabetic antenatal clinic and then collecting the placentae resulted in a long individual study period.
2. Small sample size in the groups due to long individual study period.
3. Lost to follow up 19 patients’ in our study in-spite of vigorous counseling at each antenatal visit.

## Notes

***Source of funding: ***Self and Funding Committee, Dow University of Health Sciences.

## Notes

***Conflict Of Interest Notification Page: ***This study was done as part of requirement of M Phil in Basic Medical Sciences in pharmacology at Dow University of Health Sciences. Financial burden was taken by RA, Dow University, Administration of Lyari General Hospital and Patients themselves at private obstetric clinics. Informed written consent was taken from all the patients. This study was approved by funding committee and institutional ethics review board of Dow University of Health Sciences (IRB, DUHS).
